# A Distinct Instance of Palindromic Rheumatism Disguised as Polymyalgia Rheumatica

**DOI:** 10.7759/cureus.61644

**Published:** 2024-06-04

**Authors:** Omer Farooq, Mashal Awais, Tijin Mathew, Nabeel A Siddiqui, Justin G Hovey

**Affiliations:** 1 Internal Medicine, Southeast Health Medical Center, Dothan, USA; 2 Internal Medicine: Pediatrics, Alabama College of Osteopathic Medicine (ACOM), Dothan, USA

**Keywords:** joint pain and stiffness, oral corticosteroids, seronegative rheumatoid arthritis, palindromic rheumatism, polymyalgia rheumatica

## Abstract

In this case report, we highlight a rare case of palindromic rheumatism (PR) presenting as polymyalgia rheumatica (PMR). Many challenges and complexities are associated with diagnosing and treating PR. Literature reviews showed only a few case reports of this unique presentation. PR has a distinct presentation that often goes unnoticed and is misinterpreted by medical professionals. A more thorough clinical approach is required to identify and treat this condition. We hope sharing such uncommon cases will help the medical community better understand PR and develop improved diagnostic and therapeutic options. This case also demonstrates the need for further research to better understand the pathogenesis of this uncommon condition.

## Introduction

In 1928, Philip S Hench, MD of the Mayo Clinic in Rochester, USA, identified the first case of palindromic rheumatism (PR). He encountered a 21-year-old female with recurring episodes of pain and edema in multiple joints, lasting 12 to 36 hours, with only a single joint affected at a time. Hench and Edward Rosenberg saw numerous comparable clinical appearances and published them in 1944. They noted that these attacks were unexpected, with periarticular/para-articular inflammation present. Hench and Rosenberg distinguished PR from rheumatoid arthritis (RA) based on clinical/remitting trends [1]. The clinical and imaging characteristics of PR indicate significant variations from RA and underlying molecular differences between the two disorders. PR's actual nature is uncertain; it may be regarded as a separate illness, an early stage of RA, or merely pre-RA syndrome. The genetic predisposition of PR is the same as RA [1]. PR has the potential to develop into a chronic rheumatic disease.

The observation that most patients with PR have RA-related autoantibodies and that many eventually develop RA has led to PR often being viewed as a relapsing-remitting variant of RA [2]. PMR is an inflammatory rheumatic condition characterized clinically by aches and morning stiffness. Symptoms mainly involve the shoulders, hip girdle, and neck. It may be associated with giant cell arteritis [3]. In contrast, PR presents with flares of joint pain and periarticular stiffness. Clinical symptoms of PMR typically include fatigue, weakness, mild fever, shoulder and hip girdle pain, and stiffness. It's crucial to differentiate between various illnesses like PMR and seronegative rheumatism, which can present with intermittent patterns of arthritis. This case report delves into a rare instance of palindromic rheumatism (PR) and outlines its diagnostic workup. Steroids, although infrequently employed in PR management, play a pivotal role in this particular case. Furthermore, this report elucidates the judicious use of steroids alongside nonsteroidal anti-inflammatory drugs (NSAIDs) and disease-modifying agents as effective measures in controlling PR flares.

## Case presentation

A 69-year-old male patient presented at Southeast Health Clinic, Dothan, USA, in December 2022 with complaints of bilateral hip pain, shoulder pain, and fatigue. Physical exam findings were positive for tender spots on the shoulder girdle. He was initially diagnosed with PMR, and the patient commenced treatment with prednisolone at a dosage of 1 mg/kg administered in divided doses twice daily for one month, which was subsequently tapered off. The patient underwent close monitoring every three months, and within 13 months, there was an evolution of his symptoms, prompting concerns of RA. These symptoms included bilateral joint pain involving his wrists, knees, proximal interphalangeal joints, and simultaneous periarticular muscle stiffness. The physical exam findings were positive for motion restriction of bilateral knees and wrists due to pain, redness, and swelling.

**Figure 1:**
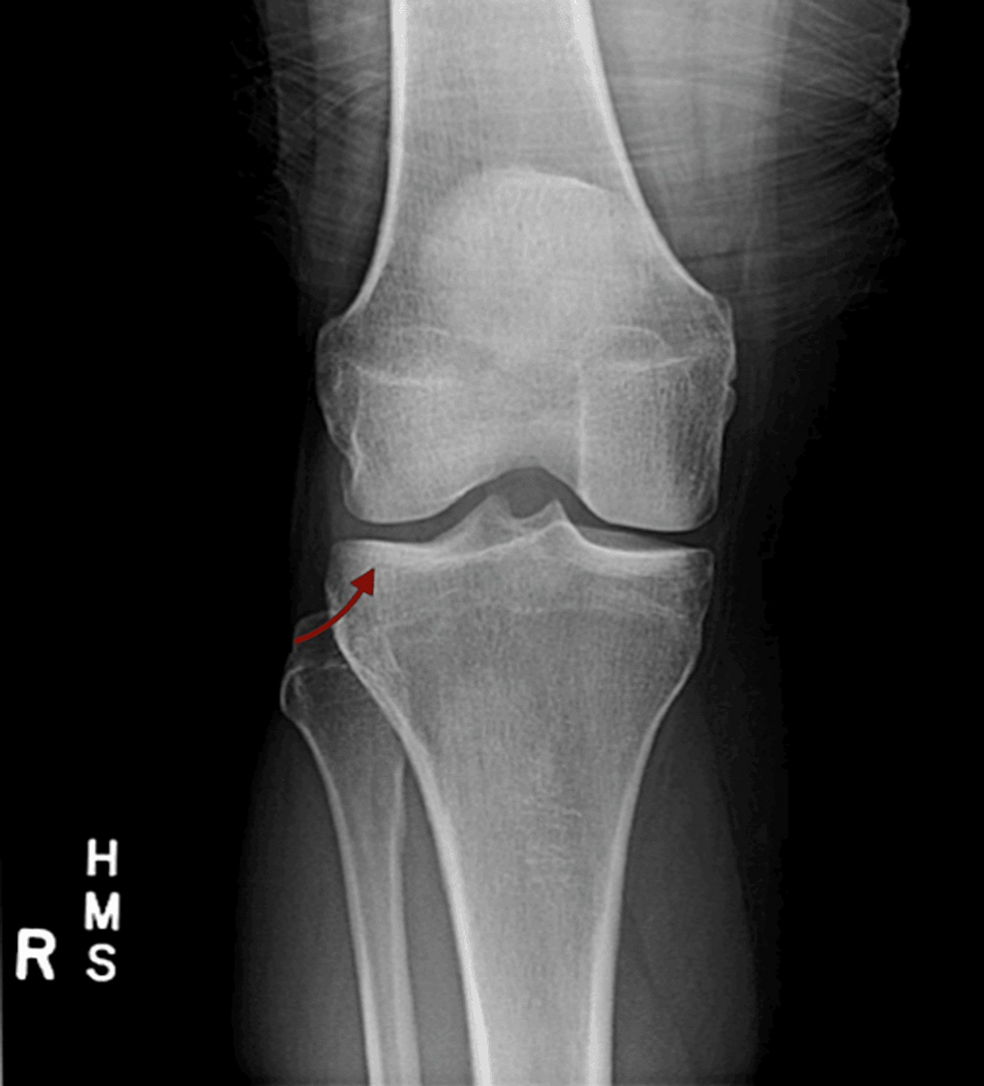
Posteroanterior view X-ray of the right knee Arrow indicates no concerning findings for seronegative rheumatoid arthritis (RA) such as narrowing of joint space and erosion of joints.

His clinical presentation and symptoms strongly suggested seronegative RA. Consequently, he commenced treatment with hydroxychloroquine at a dosage of 200 mg orally twice daily for four months. Subsequently, he was initiated on methotrexate at a dosage of 15 mg orally once weekly for three months. He could not tolerate either medication due to side effects, including generalized rash and hair loss. He experienced recurrent symptoms of arthritis and muscular stiffness, with periods of no symptoms in between. Joint X-rays were done to exclude seronegative RA, and they were negative for findings like narrowing of joint space or erosion of joints, as shown in Figure 1.

PR was suspected based on clinical symptoms in the form of flares and studies such as joint X-rays that ruled out seronegative RA. He was referred to a rheumatologist for further investigations. Despite an extensive autoimmune workup, including testing for rheumatic factor, antinuclear antibody, anti-double-stranded DNA antibody, anticitrullinated peptide antibody, anti-Sjögren’s syndrome-related antigen A, anti-Sjögren’s syndrome-related antigen B, and antineutrophil cytoplasmic antibody, all results were negative. Additionally, skeletal imaging of the affected joints showed no evidence of destructive changes. Notably, the patient exhibited a positive response to corticosteroids during acute episodes. Based on these findings, the diagnosis of PR was confirmed. Currently, the patient encounters approximately 10 flares annually, yet his symptoms are effectively managed with prednisolone at a dosage of 1 mg/kg administered in divided doses twice daily for five days, as required. The patient maintains regular follow-up appointments with the rheumatology clinic, scheduling visits every eight to 10 weeks. Despite his condition, he continues to lead an active lifestyle.

## Discussion

PR is an articular and periarticular inflammation lasting from a few hours to several days and resolving spontaneously. It can lead to an increase in inflammatory markers during relapsing episodes. The most commonly involved joints are the fingers, wrists, and knees, but other joints can also be involved [2]. Diagnosing PR poses a formidable clinical challenge due to the transient nature of inflammatory signs, often subsiding during the patient's clinic visit. Diagnostic criteria require the following: 1) Recurrent attacks of sudden-onset mono or polyarthritis or of peri-articular tissue inflammation, lasting from a few hours to weeks; 2) Verification by a physician of at least one attack; 3) Subsequent attacks in at least three different joints; 4) Exclusion of other forms of arthritis [4].

It is noteworthy that approximately one-third of individuals initially presenting with PR ultimately progress to RA [5]. Therefore, close surveillance of patients with PR is warranted for progression to RA [2]. Moreover, no laboratory or clinical characteristics enable early distinction between PMR and RA with PMR-like onset. Patients with PR typically lack the radiological findings of RA, like joint erosion and joint narrowing.

The treatment of this condition presents a unique challenge, primarily relying on clinical judgment and expertise. Non-steroidal anti-inflammatory drugs (NSAIDs) can serve as a treatment option for managing acute episodes of this condition. Disease-modifying anti-rheumatic drugs can be used to reduce symptoms and flare-ups. The response of PR patients to hydroxychloroquine provides evidence for the potential link between PR and its role as an initial manifestation of RA [6]. In a study of 113 PR patients, hydroxychloroquine lowered the risk of chronic rheumatic diseases by 20%, indicating its potential as a preventive treatment for severe rheumatic conditions [7].

As shown in our case, PMR symptoms are distinct from PR symptoms due to the disease's flare-like nature and various joint pains, which set them apart from the latter's typical presentation of fatigue and pain in the shoulder and hip girdles. PMR usually does not impact the knee joint. PR can be distinguished from seronegative RA by obtaining a thorough history, performing baseline testing, such as joint X-rays, and looking for abnormalities such as narrowing of the joint space and joint erosion. NSAIDs and corticosteroids can be used to treat PR symptoms, much as in our case study. It is not required for the patient to take steroids continuously; instead, steroids can be administered to reduce their symptoms during flare-ups. Table 1 shows the differences between PR, PMR, and seronegative rheumatic disease.

**Table 1:** Differences between PR, PMR, and seronegative rheumatic disease PR = Palindromic rheumatism; PMR = Polymyalgia rheumatica; RF = Rheumatoid factor; Anti-CCP = Anti-cyclic citrullinated peptide; ESR = Erythrocyte sedimentation rate; CRP = C-reactive protein; MCP = Metacarpophalangeal

PR	PMR	Seronegative Rheumatic Disease
Recurrent, self-limiting joint inflammation, often affecting one or a few joints.	Pain and stiffness, typically in shoulders, neck, and hips.	Heterogeneous group; absence of specific autoantibodies (RF, anti-CCP).
Episodes last hours to days and are reversible with no permanent joint damage between attacks.	Persistent symptoms lasting weeks to months.	Variable, depending on the specific disease within the group.
It can occur at any age.	It primarily affects older adults.	It can occur across various age groups.
Not always consistently elevated.	Elevated inflammatory markers (ESR, CRP).	Variable: some conditions may have elevated markers.
Monoarticular with Hand predominance, especially MCP joints	Shoulders, neck, hips; may involve other areas.	Variable; may involve peripheral joints, spine, or entheses.
One or a few joints during episodes.	Shoulders, neck, hips; may involve other areas.	Variable; may involve peripheral joints, spine, or entheses.
Generally seronegative (lack of RF or anti-CCP).	Seronegative for RF and anti-CCP.	The absence of specific autoantibodies is a defining feature.

## Conclusions

This case report emphasizes the significance of examining PR as a possible diagnosis in individuals with symptoms similar to PMR. It demonstrates the difficulties in distinguishing between these conditions due to overlapping clinical features and emphasizes the importance of a thorough clinical approach and differential diagnosis. PR patients usually do not exhibit radiological signs of seronegative RA or RA such as joint space narrowing and erosion of the joints. Strong indicators of palindromic rheumatism include relapsing-remitting joint pain, positive response to steroids, and lack of radiological findings for RA. The unique presentation of PR frequently eludes detection and is subject to misinterpretation by healthcare practitioners, necessitating a more diligent clinical approach to recognize and appropriately manage this condition. In addition to disease-modifying agents, steroids can also serve as a treatment option for managing flare-ups of PR, similar to other NSAIDs.

We hope this case improves the medical community's knowledge of PR and helps with the early detection of afflicted persons. More studies are necessary to clarify the underlying pathophysiology of PR and improve diagnostic and treatment strategies. In summary, this case highlights the significance of ongoing research endeavors, interdisciplinary cooperation, and clinical vigilance in augmenting our comprehension and handling of PR.
